# American Association of Obstetricians and Gynecologists

**Published:** 1888-10

**Authors:** 


THE AMERICAN ASSOCIATION OF OBSTETRICIANS AND GYNECOLOGISTS.—This Association was organized at Niagara Falls in the Spring of the present year, and held its first meeting in Washington, at the time of meeting of the Congress of American Physicians and Surgeons, September 18th and 20th ult. We are pleased to note the success of this meeting as shown by the scientific and practical excellence of the papers read, and the discussion thereof.

The Society is composed, largely, of representatives of the best and most progressive element of the medical profession who are specially interested in the study and practice of Obstetrics and Gynecology, and many of them, although comparatively young men, are well known both in America, and abroad, by their scientific work already accomplished.

